# Obstructive laryngeal schwannoma in a young female

**DOI:** 10.1186/s12957-014-0433-1

**Published:** 2015-02-07

**Authors:** Chang-Chieh Chiu, Shah-Hwa Chou, Chun-Chieh Wu, Peir-In Liang, Ka-Wo Lee

**Affiliations:** Department of Otolaryngology, Kaohsiung Medical University Hospital, No.100, Zyou 1st Road, Kaohsiung City, 807 Taiwan; Division of Chest Surgery, Department of Surgery, Kaohsiung Medical University Hospital, No.100, Zyou 1st Road, Kaohsiung City, 807 Taiwan; Department of Respiratory Therapy, College of Medicine, Kaohsiung Medical University, No.100, Zyou 1st Road, Kaohsiung City, 807 Taiwan; Department of Pathology, Kaohsiung Medical University Hospital, No.100, Zyou 1st Road, Kaohsiung City, 807 Taiwan; Department of Otolaryngology, College of Medicine, Kaohsiung Medical University, No.100, Zyou 1st Road, Kaohsiung City, 807 Taiwan

**Keywords:** Schwannoma, Larynx, Laryngectomy, Benign neoplasm

## Abstract

Laryngeal schwannomas are rare, benign neurogenic tumors. They normally present as a slow-growing, encapsulated, submucosal mass in the supraglottic region. We describe a 20-year-old female presenting with a 2-year history of hoarseness and progressive worsening dyspnea. Fiberoptic laryngoscopy and computed tomography revealed a round, low-density submucosal mass at right false cord and arytenoepiglottic regions with glottic extension. Microlaryngoscopic biopsy and debulking for this solid tumor were performed without tracheostomy. Schwannoma was confirmed by histopathological study. However, rapidly worsening stridor occurred 2 weeks after the surgery. Fiberoptic laryngoscopy showed an exophytic tumor occupying the right hemilarynx with airway compromise. Definite complete excision of the tumor was performed by right vertical hemilaryngectomy. At 5-month follow-up, the laryngeal wound was clear without signs of recurrence. Rapid occurrence of airway obstruction after debulking and biopsy was demonstrated in this case. Vertical hemilaryngectomy was inevitable to cure this potentially life-threatening laryngeal schwannoma in this young female with postoperative serviceable voice.

## Background

Schwannomas were first described in 1908 by Verocay [1]. They are slow-growing, benign, encapsulated, submucosal tumors derived from the Schwann cells of the peripheral nervous system. About 25 to 45% of all schwannomas occur in the head and neck region, with the majority occurring in parapharyngeal spaces. Schwannomas rarely present within the larynx, and they represent 0.1 to 1.5% of all benign laryngeal tumors [2,3].

## Case presentation

A 20-year-old Taiwanese female presented with a 2-year history of hoarseness and progressive dyspnea. Submucosal bulging in the right supraglottic area was found by her ear, nose and throat physician 1 year previously, but the patient did not wish to undergo any treatment. She had never smoked and had no throat symptoms before hoarseness developed. She denied any known systemic disease. General physical examinations were unremarkable except for moderate obesity.

**Figure 1:**
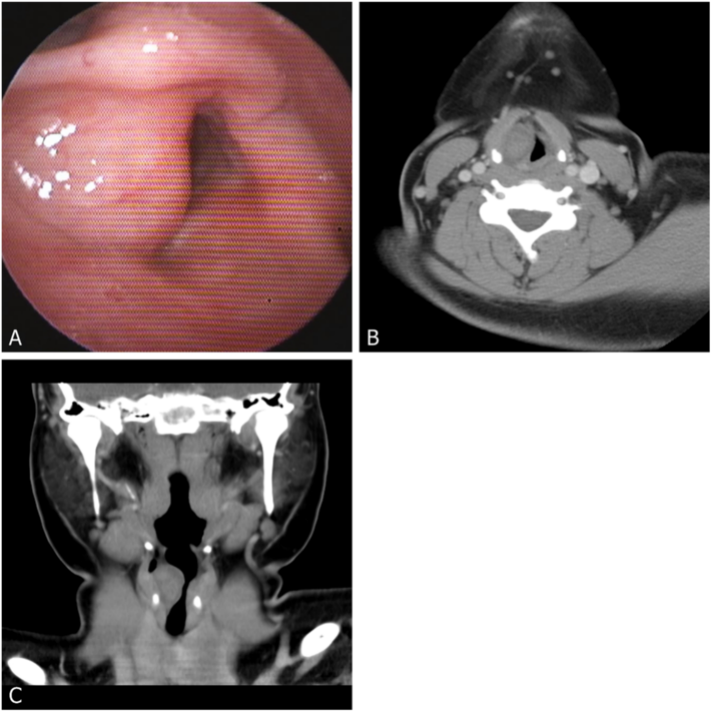
**Preoperative findings. (A)** Preoperative fiberoptic laryngoscopy revealed a submucosal bulging in the right false and true vocal folds. Preoperative contrast-enhanced computed tomography in **(B)** axial and **(C)** coronal view showed a round, slightly heterogenously enhanced mass in the supraglottic area with glottic extension.

Fiberoptic laryngoscopy revealed a round, submucosal bulging at the right false and true vocal folds (Figure 1A). The right vocal fold movement was limited and the glottic airway was narrowed. Computed tomography demonstrated a round, slightly heterogeneously enhanced mass at the right supraglottic area with glottic and possible subglottic extension, but no evidence of cartilaginous destruction was found (Figure 1B,C). A benign submucosal cystic lesion was initially impressed.

**Figure 2:**
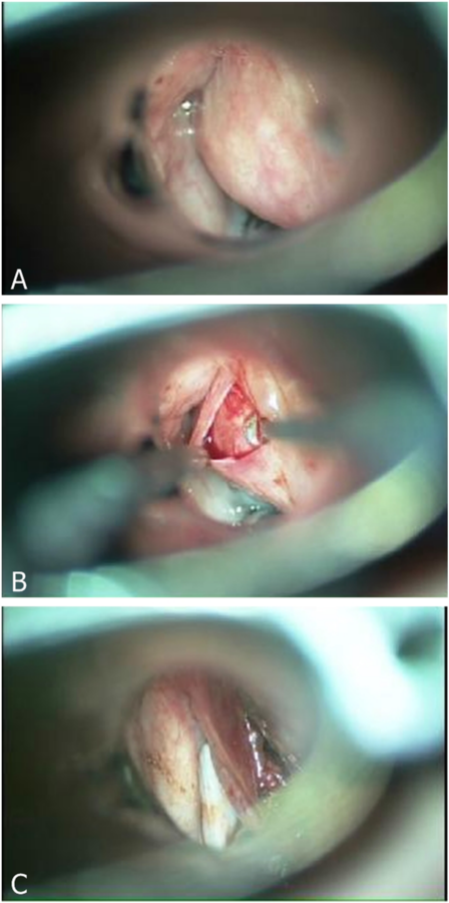
**Intraoperative findings of transoral CO**_**2**_ **laser microsurgery. (A)** A submucosal mass obstructing the view of right vocal fold was found via direct laryngoscopy. **(B)** The mucosa overlying the tumor was elevated, revealing a well-encapsulated tumor. The tumor was adhered to the underlying cartilage. **(C)** With CO_2_ laser, the tumor was removed as much as possible.

**Figure 3:**
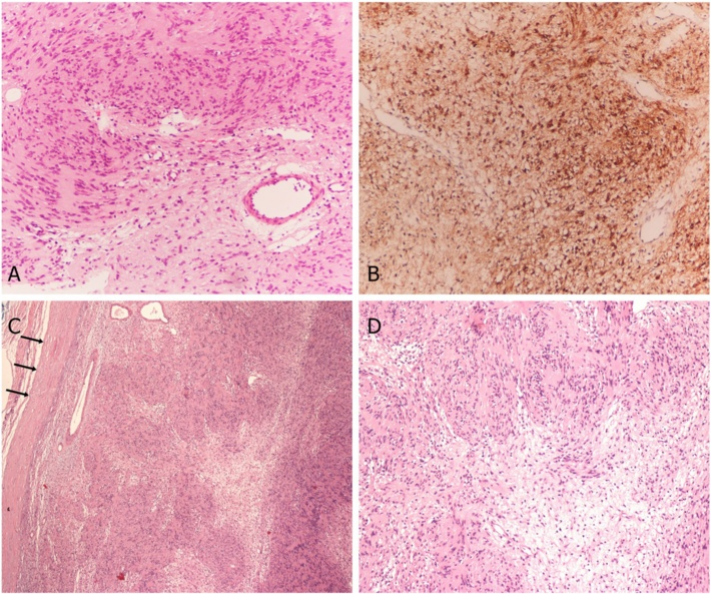
**Histopathological findings of transoral CO**_**2**_ **laser microsurgery and vertical hemilaryngectomy. (A,B)** Transoral CO_2_ laser microsurgery: **(A)** the histopathological specimen shows biphasic pattern of compact hypercellular Antoni A and myxoid hypocellular Antoni B areas (hematoxylin-eosin stain, magnification × 200); **(B)** the schwannoma cells showed strong immunoreactivity for S-100 protein. **(C,D)** Vertical hemilaryngectomy: microscopically, the specimen of vertical laryngectomy showed characteristic findings of schwannoma without surrounding tissue or vascular invasion (black arrow in **(C)**: capsule) (hematoxylin-eosin stain, **(C)** magnification × 40, **(D)** magnification × 100).

During microlaryngoscopic examination, an elastic submucosal tumor with involvement of right false and true vocal folds was found. The surgeon raised a mucosal flap and blunt dissected along the capsule of the solid tumor. Total removal of the lesion was impossible, hence biopsy and debulking with CO_2_ laser were performed (Figure 2). The patient was extubated immediately after the procedure, and discharged with oral antibiotics on the next day. The histopathological study showed schwannoma with intense immunoreactivity for protein S-100 and negative for SMA (Figure 3A,B).

**Figure 4:**
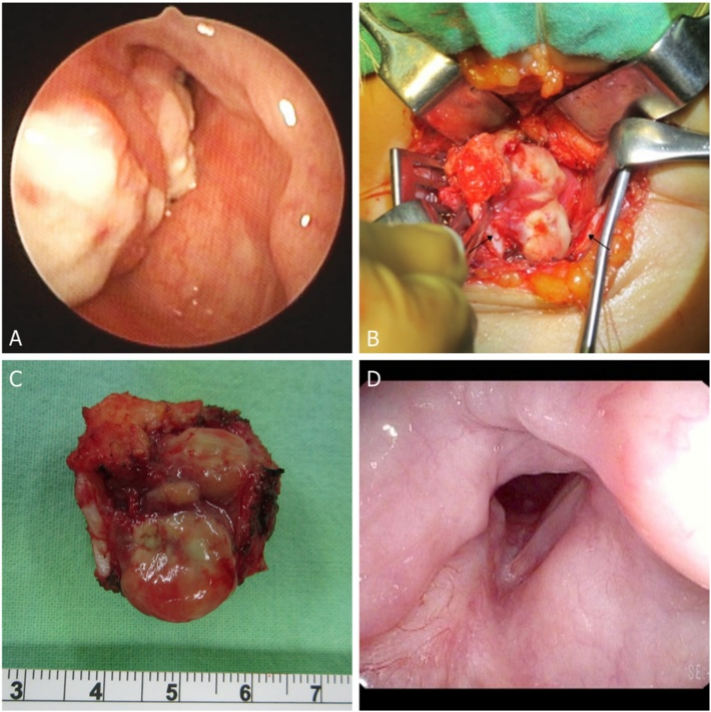
**Pre- and intraoperative findings of vertical hemilaryngectomy. (A)** Fiberoptic laryngoscopy revealed space-occupying tumor in the right hemilarynx with partial airway obstruction 2 weeks after transoral CO_2_ laser debulking. **(B)** Intraoperative findings of vertical hemilaryngectomy: an exophytic tumor extending from supraglottis to subglottic region was found. Black arrow: cutting edge of laryngofissure. **(C)** Resected tumor showing glottic and subglottic extension. **(D)** Laryngoscopic view 5 months after vertical hemilaryngectomy.

However, the patient returned to our clinic with dyspnea and inspiratory stridor 2 weeks after the debulking and biopsy procedures. Fiberoptic laryngoscopy revealed a space-occupying tumor in the right hemilarynx with partial airway obstruction (Figure 4). Emergent tracheotomy was performed for airway protection. Definite total extirpation of the tumor was executed by right lateral hemilaryngectomy. The tumor was removed completely with the underlying thyroid cartilage with well-delineated margins under direct vision. Macroscopically, there was a 2 × 2.5 cm, yellowish-colored exophytic tumor extending from the supraglottis to glottis and subglottic regions. Histological evaluation showed biphasic pattern of compact hypercellular Antoni A and myxoid hypocellular Antoni B areas without nuclear pleomorphism or mitotic activity (Figure 3C,D). Inflammatory change was found at the previous operation wound. The diagnosis of schwannoma was again confirmed. No laryngeal stent was used and the postoperative period was uneventful. She did not receive postoperative chemotherapy or radiotherapy. Follow-up visit 5 months after definitive surgery revealed clear laryngeal wound without signs of recurrence or stenosis. The patient had a serviceable voice postoperatively.

## Discussion

Benign neurogenic tumors are rare in the larynx and comprise only about 0.1 to 1.5% of all benign laryngeal tumors [4]. Two different types of neurogenic tumor of the larynx have been described: schwannoma and neurofibroma. Schwannomas deriving from perineural Schwann cells grow extrinsically to their parent nerve fascicles and may develop along any somatic or sympathetic nerve in the body (except the olfactory and optic nerves that lack Schwann’s cells) [5]. By contrast, neurofibromas originate from perineural fibrocytes, involving nerve fibers and sheath cells, and they are usually intertwined with the nerve trunk [3]. This differentiating characteristic is important from the surgical viewpoint because surgical removal of a tumor from the originating nerve is theoretically possible in schwannomas, but impossible in neurofibromas [4]. Neurofibromas are encountered more frequently in neurofibromatosis. Malignant transformation is reported in 10% of neurofibromas while it is very uncommon in schwannomas [3].

Laryngeal schwannomas most commonly arise in the supraglottic region (that is, in the aryepiglottic fold or the true or false vocal cords), but they can develop in the subglottic region in extremely rare cases [6,7]. Schwannomas occur at any age with an increased incidence in fourth and fifth decades of life, predominantly in women [3,8]. The internal branch of the superior laryngeal nerve was presumed to be the origin of the tumors [9].

The clinical symptoms are related to the mass effect of a slowly growing lesion in the larynx: the patient gradually develops hoarseness, globus sensation and dysphagia over years rather than weeks or months [5]. As the tumor expands, it may cause dyspnea and stridor. Some patients complain about dyspnea in the supine position, which seems to be associated with the location of the lesion. Asphyxial death due to laryngeal schwannoma has also been reported [8].

The diagnostic work-up in such cases should include fiberoptic laryngoscopy, image study, and histological biopsy. On laryngoscopy, the characteristic finding is a round submucosal bulge confined to the aryepiglottic fold or false vocal cord. This was in concordance with our patient. It may obstruct the view of the laryngeal inlet or result in reduced mobility of the vocal cord. [5] Image studies could provide more information to differentiate laryngeal submucosal tumors. On computed tomography scans, schwannomas appear as submucosal, well-defined, hypodense masses, without destruction of adjacent structures [5]. A heterogeneous contrast enhancement was also described. In the present case, planes between the tumor and the thyroid cartilage seemed to be poorly defined, which was compatible with our operative findings. On magnetic resonance imaging scans, the lesions appear isointense to slightly hyperintense in T1-weighted sequences, hyperintense in T2-weighted sequences and hyperintense with gadolinium enhancement [10]. Differential diagnosis includes laryngeal cysts, laryngoceles, adenomas, chondromas and malignant tumors [11].

Since endoscopic assessment and imaging studies cannot rule out malignancy, the definite diagnosis can only be made histologically. Enger and Weiss established three histological criteria for the diagnosis of schwannoma: the presence of a capsule, the presence of a stromal Antoni A (compacted, bipolar cells with nuclei arranged in a palisade form) and/or Antoni B (loosely arranged spindle cells within a myxoid matrix) histological pattern, and positive staining with S-100 (characteristic of Schwann cells) [3]. All of these three features were seen in our case.

To prevent recurrence, treatment of laryngeal schwannomas is based on complete surgical resection. In addition, preservation of laryngeal function and protection of the laryngeal mucosa from surgical injury are the keys to better surgical outcome.

Surgical intervention should be planned according to the symptoms of each patient, as well as the location and extent of the tumor. For smaller tumors with adequate endolaryngeal exposure, transoral CO_2_ laser microsurgery can be a reasonable treatment option [3,12]. Moreover, transoral robotic surgery-assisted excision of schwannoma in the supraglottic larynx has been reported recently [13]. Larger tumors or recurrent disease may require a tracheostomy followed by an external approach; for example, lateral thyrotomy, lateral pharyngotomy, or laryngofissure technique [5].

Rapid return of symptoms after initial biopsy or surgery has been described for supraglottic laryngeal schwannomas, and these patients were eventually managed with transoral CO_2_ laser microsurgery or median thyrotomy [5,11]. In this present case, severe adhesion was found between the tumor and underlying cartilage. Endoscopic debulking with CO_2_ laser and biopsy could only be used initially due to the location and size of the tumor. The reasons for the rapid enlargement and return of obstructive symptom within 2 weeks in our particular patient were considered to be tumor inflammation and infection. Besides, malignancy needed to be ruled out in this aggressive clinical presentation. Hence, vertical hemilaryngectomy was inevitable as the definitive surgery. Extreme care was used to preserve as much normal endolaryngeal mucosa as possible in order to facilitate favorable clinical outcome in our patient.

## Conclusions

Laryngeal schwannomas are rare, benign neurogenic tumors. In this young female, vertical hemilaryngectomy was inevitable for her potential life-threatening, infected, large supraglottic schwannoma with glottic and subglottic involvement.

## Consent

Written informed consent was obtained from the patient for publication of this case report and any accompanying images. A copy of the written consent is available for review by the Editor-in-Chief of this journal.
